# Retailer Compliance With Tobacco 21 in New Jersey, 2019-2020

**DOI:** 10.1001/jamanetworkopen.2022.35637

**Published:** 2022-10-05

**Authors:** Mary Hrywna, Amanda Y. Kong, Christopher Ackerman, Shawna V. Hudson, Cristine D. Delnevo

**Affiliations:** 1 Rutgers Center for Tobacco Studies, Rutgers Biomedical and Health Sciences, New Brunswick, New Jersey; 2 Department of Health Behavior, Society and Policy, Rutgers School of Public Health, Piscataway, New Jersey; 3 Department of Family and Preventive Medicine, University of Oklahoma Health Sciences Center, Oklahoma City; 4 TSET Health Promotion Research Center, Stephenson Cancer Center, University of Oklahoma Health Sciences Center, Oklahoma City; 5 Department of Family Medicine and Community Health, Rutgers Robert Wood Johnson Medical School, New Brunswick, New Jersey

## Abstract

This cross-sectional study assesses retailer compliance with the Tobacco 21 law in New Jersey during 2019 and 2020.

## Introduction

Before passage of the federal law in 2019, several states increased the tobacco age of sale to 21 years. The extent to which Tobacco 21 (T21) will reduce underage access depends on high retailer compliance. The limited research on age of sale compliance is mixed but suggests that T21 is not enforced uniformly.^1,2,3^ Moreover, current compliance protocols have methodological limitations that limit validity.^4^ A familiarity protocol that mimics actual purchasing behaviors is recommended, including repeated visits to the same store with the same buyer.^5^ We conducted a tobacco purchase study in New Jersey, a T21 state since 2017, to assess retailer compliance and its correlates.

## Methods

This cross-sectional study was determined to be non–human participants research by the Rutgers Health Sciences institutional review board; thus, the need for informed consent was waived in accordance with 45 CFR §46. This study followed the Strengthening the Reporting of Observational Studies in Epidemiology (STROBE) reporting guideline.

Detailed study methods are in the eAppendix in the Supplement. In brief, we drew a random sample of 100 licensed tobacco retailers, audited for product availability, and removed 14 retailers for liquor sales, safety concerns, permanent closure, or no sales of tobacco products. Of the remaining 86 retailers, 100% sold cigarettes, 79.1% sold cigars, and 45.3% sold e-cigarettes. Repeated purchase attempts for each product were then made by 5 buyers (aged 18-20 years) between August 2019 and March 2020 in all stores. Buyers recorded carding (ie, age verification) and purchase attempt outcomes at each visit. Because buyers attempted a single product purchase at each visit, a store could be visited up to 15 times. Thus, we used general estimating equations, and a buyer-level fixed effect was included in unadjusted and adjusted models. Data were analyzed in June 2021 using SAS statistical software version 9.4 (SAS Institute).

## Results

Of total visits, underage buyers were carded in 65.9% of visits and successfully purchased products in 42.3% of visits, but if carded, only 14.0% of visits resulted in a successful product purchase (Table 1). Adjusted models indicated that nonchain convenience stores (adjusted odds ratio [aOR], 0.18; 95% CI, 0.08-0.40) and gas kiosks (aOR, 0.24; 95% CI, 0.10-0.59) had lower odds of carding compared with chain convenience stores (Table 2). Greater tobacco retailer density was also associated with lower odds of carding (aOR, 0.80; 95% CI, 0.64-0.98). Drugstores had lower odds (aOR, 0.20; 95% CI, 0.07-0.61) of successful purchases compared with chain convenience stores. Failure to card was associated with higher odds of a successful tobacco purchase (aOR, 313.36; 95% CI, 122.18-803.66). Models assessing the odds of a successful purchase after a successful identification check indicated similar associations, though none were significant.

**Table 1.** Description of ID Checks and Successful Purchases by Tobacco Product Type and Store Type, New Jersey, 2019-2020

Explanatory variable	Visits, No. (%)		
	Overall (N = 780)		Successful purchase among visits where ID was checked (n = 514)
	ID checked	Successful purchase	
Total visits	514 (65.9)	330 (42.3)	72 (14.0)
Product type			
Cigarettes	215 (66.2)	138 (42.5)	29 (13.5)
Cigars	188 (61.2)	142 (46.3)	25 (13.3)
e-Cigarettes	111 (75.0)	50 (33.8)	18 (16.2)
Store type			
Chain convenience	133 (84.7)	41 (26.1)	17 (12.8)
Convenience (nonchain)	187 (51.0)	212 (57.8)	36 (19.3)
Gas kiosk only	44 (56.4)	38 (48.7)	6 (13.6)
Drugstore	67 (87.0)	11 (14.3)	2 (3.0)
Other (eg, grocery, dollar stores)	83 (82.2)	28 (27.7)	11 (13.3)

Abbreviation: ID, identification.

**Table 2.** Associations of Retailer, Purchase, and Neighborhood Characteristics With Identification Checks and Successful Purchases for 780 Store Visits, New Jersey, 2019-2020^a^

Explanatory variables	Identification check (yes vs no)		Successful purchase (yes vs no)	
	OR (95% CI)	aOR (95% CI)	OR (95% CI)	aOR (95% CI)
Product type attempted for purchase				
Cigarettes	1 [Reference]	1 [Reference]	1 [Reference]	1 [Reference]
Cigars	0.81 (0.61-1.08)	0.80 (0.57-1.11)	1.28 (0.99-1.65)	1.11 (0.62-1.98)
e-Cigarettes	0.97 (0.73-1.29)	0.96 (0.69-1.33)	1.06 (0.74-1.51)	0.81 (0.39-1.69)
Store type				
Chain convenience	1 [Reference]	1 [Reference]	1 [Reference]	1 [Reference]
Convenience (nonchain)	0.18 (0.08-0.37)	0.18 (0.08-0.40)	4.47 (2.22-9.00)	1.62 (0.71-3.69)
Gas kiosk only	0.28 (0.12-0.64)	0.24 (0.10-0.59)	2.15 (0.88-5.25)	0.88 (0.21-3.63)
Drugstore	1.07 (0.23-5.00)	1.33 (0.25-7.07)	0.53 (0.13-2.10)	0.20 (0.07-0.61)
Other (eg, grocery, dollar stores)	0.81 (0.30-2.15)	0.77 (0.27-2.18)	1.14 (0.48-2.73)	0.97 (0.35-2.63)
Clerk asked for identification (no vs yes)	NA	NA	302.90 (127.24-720.92)	313.36 (122.18-803.66)
Tobacco retailers per 1000 people	0.85 (0.67-1.08)	0.80 (0.64-0.98)	1.14 (0.92-1.43)	1.10 (0.88-1.37)

Abbreviations: aOR, adjusted odds ratio; NA, not applicable; OR, odds ratio.

^a^ For all models, generalized estimating equations were used to account for the nesting of visits to tobacco retailers within tobacco retailers, and a buyer-level fixed effect was added. Unadjusted models include each explanatory variable separately, whereas adjusted models include all explanatory variables.

## Discussion

Although previous research has documented tobacco retail sales violations, fewer studies have examined age verification and illegal tobacco sales in the context of T21. In this cross-sectional study using a familiarity protocol, underage buyers successfully purchased tobacco more than 40% of the time. Carding may be the most important deterrent to underage sales given the strength of its association with purchasing. However, even when identification was checked, underage purchases still occurred. Carding was associated with store type, consistent with prior research suggesting that nonchain stores may provide greater youth access.^1^ In addition, to our knowledge, this is the first study to explore tobacco retailer density in the context of T21. Further investigation into community and contextual factors, such as tobacco retailer density and tobacco product diversity, should be considered when assessing retailer compliance.

This study has limitations. First, the study may be underpowered to detect some associations, particularly for product type, where there were fewer store visits for noncigarette products. Second, generalizability is limited to New Jersey, and T21 implementation will likely vary by state. Third, our sample did not include vape shops. Fourth, a small proportion of buy attempts occurred after enactment of federal T21 but before Food and Drug Administration enforcement. Despite these limitations, our findings support the need for compliance check inspection protocols that more closely mimic the behaviors of young people.^4,5^ Federal and state authorities should enforce rigorous age verification for all tobacco sales and consider measures to regulate tobacco retailer density to further reduce underage access to tobacco.

## Notes

Supplement. **eAppendix.** Supplemental Methods

## References

[1] Silver D, Macinko J, Giorgio M, Bae JY, Jimenez G. Retailer compliance with tobacco control laws in New York City before and after raising the minimum legal purchase age to 21. Tob Control. 2016;25(6):624-627. doi:10.1136/tobaccocontrol-2015-052547 26585707

[2] Zhang X, Vuong TD, Andersen-Rodgers E, Roeseler A. Evaluation of California’s ‘Tobacco 21’ law. Tob Control. 2018;27(6):656-662. doi:10.1136/tobaccocontrol-2017-054088 29440328 PMC6252367

[3] Roberts ME, Klein EG, Ferketich AK, . Beyond strong enforcement: understanding the factors related to retailer compliance with Tobacco 21. Nicotine Tob Res. 2021;23(12):2084-2090. doi:10.1093/ntr/ntab093 33982115 PMC8757316

[4] Lee JGL, Gregory KR, Baker HM, Ranney LM, Goldstein AO. “May I buy a pack of Marlboros, please?” a systematic review of evidence to improve the validity and impact of youth undercover buy inspections. PLoS One. 2016;11(4):e0153152. doi:10.1371/journal.pone.0153152 27050671 PMC4822877

[5] Landrine H, Klonoff EA. Validity of assessments of youth access to tobacco: the familiarity effect. Am J Public Health. 2003;93(11):1883-1886. doi:10.2105/AJPH.93.11.1883 14600057 PMC1448067

