# Supplementary material for: Retailer Compliance With Tobacco 21 in New Jersey, 2019-2020
Source: JAMA Netw Open. 2022 Oct 5;5(10):e2235637. doi: 10.1001/jamanetworkopen.2022.35637 (PMC9535538; doi:10.1001/jamanetworkopen.2022.35637)
Supplement: Supplement. — eAppendix. Supplemental Methods [file jamanetwopen-e2235637-s001.pdf]

Supplemental Online Content

Hrywna M, Kong AY, Ackerman C, Hudson SV, Delnevo CD. Retailer compliance with Tobacco 21 in New Jersey, 2019-2020. *JAMA Netw Open*. 2022;5(10):e2235637. doi:10.1001/jamanetworkopen.2022.35637

eAppendix. Supplemental Methods

This supplemental material has been provided by the authors to give readers additional information about their work.

© 2022 Hrywna M et al. *JAMA Network Open*.

eAppendix. Supplemental Methods

Sample

The sampling frame in New Jersey included all licensed tobacco retailers in municipalities within a 25-mile radius of New Brunswick, New Jersey. In 2019, there were 4011 tobacco retailers within the selected area. We identified and removed 529 liquor stores that from the sampling frame. We then randomly sampled 50 retailers from high population density municipalities (n=1884) and 50 retailers from low population density municipalities (n=1598). Prior to fielding, research staff completed store audits in person to confirm that stores were open and assessed availability of all tobacco products, after which 14 stores were removed from the sample because of permanent closure (4), bar/liquor stores (4), safety concerns (3), or tobacco no longer for sale (3), leaving a final sample of 86 licensed tobacco retailers.

Covert buyers

We recruited and trained five covert buyers between the ages of 18 and 20. We attempted to recruit a racially, ethnically, and gender diverse team of buyers. The team of buyers was ultimately comprised of five individuals: a non-Hispanic White male aged 19, a Black or African American male aged 19, a non-Hispanic White female aged 20, a Hispanic or Latino White female aged 19, and a non-Hispanic White female of Middle Eastern ethnicity aged 19. Each buyer visited all the stores, individually, to attempt purchases of cigarettes, cigars, and e-cigarettes on separate occasions.

Data collection

In separate visits, each covert buyer was assigned to attempt purchases for each product (e.g., cigarettes, cigar/cigarillos, e-cigarettes) in every store where the product was available. Federal law requires carding anyone appearing under age 27 who attempts to purchase tobacco. If a store clerk asked buyers about age or more specifically, if they were 21, the buyers were instructed to say, “Do you want to see my ID?” Our buyers were instructed not to lie about age and to show their legal driver’s license if asked for ID. In addition to providing birthdate, a New Jersey driver’s license is printed on the vertical axis if the license holder is under 21. Buyers repeated purchase attempts for each available product in all stores, regardless of the prior outcome for a product.

Measures

For each store visit, buyers recorded what tobacco product they attempted to purchase (i.e. cigarettes, cigars, or e-cigarettes). Additionally, during our availability audit, each store type was classified (i.e. chain convenience, convenience [non-chain] bodega, drug store, gas kiosk only, other [such as discount dollar stores]). Stores classified as chain convenience were typically a franchise or corporate owned in multiple locations with similar inventory and layout, often licensed under a parent company name like Quick Chek or WaWa while non-chain convenience or bodegas were smaller, independently owned grocery/convenience stores with less diverse product inventory and typically licensed under an individual name. Drug stores included all pharmacies including both chain (e.g., Walgreens, Rite-Aid) and smaller independently owned pharmacies. Gas kiosks were retailers primarily selling fuel but limited in other inventory (e.g.. did not also include convenience store). General merchandisers such as grocery and dollar stores were classified as other stores.

After each store visit, buyers used a Qualtrics survey to record whether they were able to attempt the purchase, the product they attempted to purchase, whether the store clerk asked for identification when attempting to purchase including electronic verification, and whether they were successfully able to purchase. If they were able to purchase the product, they are also asked to provide the price of the product.

© 2022 Hrywna M et al. *JAMA Network Open*.
